# Toe Gangrene as the First Presenting Symptom of Essential Thrombocythemia: A Case Report

**DOI:** 10.7759/cureus.42388

**Published:** 2023-07-24

**Authors:** Mahmoud M Altayyan, Mohammad Abu-Tineh, Awni Alshurafa, Mohammed Abdulgayoom, Mohammad Afana, Khalid Ahmed, Haneen A Toba, Mohamed A Yassin

**Affiliations:** 1 Department of Medical Education, Hamad Medical Corporation, Doha, QAT; 2 Department of Medical Oncology, Hematology, and Bone Marrow Transplant, National Center for Cancer Care and Research, Doha, QAT

**Keywords:** thrombocythemia, ischemia, lower limb, thrombosis, myeloproliferative neoplasm

## Abstract

Essential thrombocythemia is a myeloproliferative neoplasm. Thrombosis and bleeding complications are common with myeloproliferative neoplasms, particularly essential thrombocythemia and polycythemia vera. Here, we report the case of a 52-year-old female who presented initially with painful toe swelling and discoloration. Initial imaging showed a small abscess. An incision and drainage, and debridement of toe dry gangrene were performed twice in two months with no improvement in her complaint and worsening discoloration, ending in a toe amputation. Two years later, the patient was referred to a hematology clinic for a high platelet count. On review of her medical records, the patient had the same numbers during the initial presentation. The patient’s condition was diagnosed retrogradely by a hematologist as essential thrombocythemia. This case sheds light on myeloproliferative neoplasm as a differential diagnosis in patients with atypical thrombosis. Thinking in such a way could have diagnosed our patient two years earlier.

## Introduction

Philadelphia-negative (BCR-negative) myeloproliferative neoplasms are classically described as polycythemia vera, myelofibrosis, and essential thrombocythemia [1]. According to the World Health Organization (WHO) classification, essential thrombocythemia (ET) is a diagnosis of exclusion for patients with a platelet count exceeding 450,000 mm3 whose bone marrow biopsy suggests the absence of reactive thrombocytosis [2-4]. Essential thrombocythemia is associated with many complications, most commonly thrombosis.

The available data estimated the annual incidence of ET in Western countries at 0.2 to 2.5 cases per 100,000 and a prevalence of 38 to 57 cases per 100,000. In the United States, the available data show that the annual incidence of ET is 2.5 per 100,000, whereas the prevalence is 24 cases per 100,000. [5-6] No recently published data have shown the real incidence and prevalence of ET, especially in the Middle East.

In this report, we describe the case of a 52-year-old female patient with toe swelling that did not improve over two months, leading to amputation. A hematologist finally diagnosed essential thrombocythemia.

In general, arterial thrombosis occurs due to preexisting atherosclerotic plaque, arterial narrowing, intraplaque hemorrhage, and local hypercoagulability. It can occur in an aneurysmal or dissected vessel. Platelet-mediated transient and occlusive thrombosis in end-arterial circulation is responsible for microvascular abnormalities (e.g., atypical and typical transient cerebral, ocular, and coronary ischemic episodes) in polycythemia vera and essential thrombocythemia, as well as erythromelalgia. Patients diagnosed with essential thrombocythemia and microvascular abnormalities exhibit lower platelet survival, higher beta-thromboglobulin, platelet factor 4, and thrombomodulin levels, and elevated urinary thromboxane B2 excretion. All of these findings suggest platelet-mediated thrombotic events [7,8]. Despite a significant correlation between myeloproliferative neoplasms and thrombosis of both the arterial and venous systems [9], many general practitioners are unaware of this link. When certain thrombosis events occur without a clear local pathology, patients must be evaluated for molecular abnormalities related to myeloproliferative neoplasms.

Most thromboses attributed to myeloproliferative neoplasms, such as essential thrombocythemia, are arterial events [9]. Less commonly, thromboses can occur in the cerebral, splanchnic, portal, mesenteric, and splenic veins, as well as the hepatic veins (i.e., Budd-Chiari syndrome) [9,10]. The reported incidence of significant thrombosis upon diagnosis varies between 9.7% and 29.4% with essential thrombocythemia.

## Case presentation

A 52-year-old female patient with no medical history presented to Hamad Hospital's emergency medicine department. A non-smoker with no history of prior hospitalization, she complained of painful swelling and discoloration in her right fifth toe lasting about four weeks. She had received a one-week course of antibiotics (she didn’t know the name of the medicine) before the first presentation with no improvement. The pain was gradual in onset, progressive, persistent, and throbbing, limiting her ambulation and waking her at night. The discoloration was black at the time of the presentation. She denied any history of other skin changes or recent trauma, and a systemic review was otherwise unremarkable.

On physical examination, she was vitally stable. A cold, mildly swollen right fifth toe was observed on her right foot. The toe had blackish discoloration at the tip that faded on the rest of the toe. The patient exhibited severe tenderness and a palpable, strong dorsalis pedis pulse with no neurological motor deficit. Otherwise, the examination was unremarkable.

The Doppler ultrasound of the right lower limb showed distal posterior tibial, peroneal, and dorsalis pedis arteries with normal waveforms in a maintained, triphasic pattern. An ultrasound of the soft tissue showed a very small (0.5 x 0.2 x 0.5 cm) hypoechoic area with edema along the tip of the fifth toe. The initial complete blood count results revealed a platelet level of 639 × 103 μL (normal range: 150 to 450 × 103/μL). Table 1 shows the blood test results.

**Table 1:** The patient's initial blood test results

	Result	Reference range
Hemoglobin	13.5 gm\dl	11.6–15 gm\dl for women
Platelets	639×10^3/*μ*L	150 to 450×10^3/*μ*L
White blood cells	11x10^3 μL	4.5 to 11.0×10^3/ μL
Red blood cells	5.9×10^6/*μ*L	(3.8–4.8)×10^6/*μ*L
Hematocrit	44.3%	36–46%
Mean corpuscular volume	75.1 fL	83–101 fL
Mean corpuscular hemoglobin	22.9 pg	27–32 pg
Mean corpuscular hemoglobin concentration	30 gm\dl	31.5–34.5 gm\dl
Red blood cell distribution width	17.4%	11.6–14.5%
Prothrombin time	13.6 seconds	9.7–11.8 seconds
International normalized ratio	1.3	<= 1.1
Activated partial thromboplastin time	35.2 seconds	24.6–31.2 seconds
Urea	4.9 mmol\L	2.8–8.1 mmol\L
Create	92 Umol\L	44–80 Umol\L
Sodium	142 mmol\L	136–145 mmol\L
Potassium	4.9 mmol\L	3.5–5.1 mmol\L
Chloride	103 mmol\L	98–107 mmol\L
Bicarb	28 mmol\L	22–29 mmol\L
Calcium	2.39 mmol\L	2.15–2.50 mmol\L
Adjusted calcium	2.39 mmol\L	2.15–2.50 mmol\L
Bilirubin	6 Umol\L	0–21 Umol\L
Total protein	72 gm\L	66–87 gm\L
Albumin	40 gm\L	35–52 gm\L
Aspartate aminotransferase	23 U\L	0–23 U\L
Alanine transaminase	31 U\L	0–33 U\L

The patient's long hospital course started with a collection incision and drainage, and debridement of dry gangrene at the toe tip. A wound culture identified *Klebsiella spp.*, for which the patient was administered intravenous cefuroxime (1,500 mg) every eight hours for 14 days based on sensitivity. On day 11 of hospitalization, as the patient was still complaining of toe pain and tenderness on examination, an MRI of the right foot was done and showed features consistent with osteomyelitis of the distal phalanx of the fifth toe with exposure of the tip of the bone. On day 15 of the hospital stay, after discussing options with the orthopedic team, the vascular surgery team, and the patient, a decision was made to amputate the right fifth toe.

During her six-week hospital stay, the patient’s platelet count ranged from a low of 639×103/μL on initial presentation to a high of 816×103/μL on day 10 (normal range: 150 to 450×103/μL).

Nearly one year after the amputation procedure, the patient visited her primary healthcare physician complaining of generalized itching. A basic investigation showed a high platelet count of 610×103/μL, so she was referred to the hematology clinic for suspicion of thrombocytosis. A peripheral smear showed giant platelets. A bone marrow aspiration and biopsy were done, along with morphology, cytogenetics, flow cytometry, and Fluorescence in situ hybridization (FISH) analyses, which showed a positive Janus kinase 2 (JAK2) V617F missense mutation. A diagnosis of essential thrombocythemia was established.

## Discussion

This is a rare presentation of toe gangrene that was initially treated without a deep evaluation of the etiology behind it, leading to a late identification of it as a first sign of essential thrombocythemia with no apparent risk factors. Typical sites of thrombosis in myeloproliferative disorders are the large arteries, though in polycythemia vera and essential thrombocythemia, thromboses can occur in atypical sites such as the cerebral venous sinuses and the splanchnic and hepatic veins [11]. Thrombosis occurs in about 20% to 50% of patients with essential thrombocythemia; yet, defining the exact risk of thrombosis in this condition remains challenging [12].

Estimates have been derived from prospective and retrospective research, and risk variables such as advancing age and history of thrombosis have been identified as key contributors. Though no correlation between platelet count and thrombotic event incidence has been established, evidence indicates that adequate control of platelet count reduces the incidence of thrombosis [12].

Aspirin can alleviate vasomotor and microvascular occlusive symptoms or signs, but evidence of its use in lessening the risk of bigger vessel thrombosis is limited. Clonal hematopoiesis in younger patients, high cholesterol, and cigarette smoking are thought to be risk factors for thrombosis [8]. Cytoreductive treatment must be administered to patients at high risk of venous or arterial thrombosis, and most patients favor hydroxyurea because of its efficacy, tolerability, and affordability [13,14].

Multiple studies have compared anagrelide, a cytoreductive therapy, with aspirin and hydroxyurea. In Anagrelide Compared with Hydroxyurea in WHO-classified Essential Thrombocythemia: The ANAHYDRET Study, results of two randomized trials concluded that anagrelide was non-inferior to hydroxyurea in preventing thrombo-hemorrhagic complications, and the anagrelide group had more cardiac events [15]. Prior studies have demonstrated that hydroxyurea plus low-dose aspirin is superior to anagrelide plus low-dose aspirin for patients with essential thrombocythemia at high risk for vascular events [16]. Interferon can be administered in resistant cases or to patients who cannot tolerate hydroxyurea. Interferon-alpha has shown therapeutic effects in polycythemia vera and essential thrombocythemia, as indicated by numerous small studies and single-arm trials [17-19]. Controlling excessive erythrocytosis and thrombocytosis, as well as vasomotor symptoms, pruritus, and splenomegaly, are among the reported positive benefits [11,20].

For our patient, after a hematologist assessment and discussion with the patient about the available treatment lines, she has been started on low-dose aspirin 100 mg, followed regularly in the hematology clinic, without any further occlusive symptoms.

## Conclusions

Essential thrombocytosis is an indolent disorder with long-term survival rates. Risk factors include arterial ischemia in those aged 60 years and older, a platelet count over 450 or a leukocyte count over 11,000 mcL (11×109/L), and a history of thrombosis. These symptoms should trigger further investigation of hematological diseases. Furthermore, the absence of local pathology with arterial thrombosis presentation might direct toward evaluation for molecular abnormalities related to myeloproliferative neoplasms after careful assessment of the patient’s clinical and laboratory findings. Thrombosis in essential thrombocythemia is a significant source of morbidity and can be reduced by maintaining a platelet count under 500,000/mcL (500×09/L).
